# Effects of Passive Hip Flexion and Extension Assistance in Patients with Peripheral Artery Disease and Healthy Individuals

**DOI:** 10.3390/s25113368

**Published:** 2025-05-27

**Authors:** Hiva Razavi, Sara A. Myers, Iraklis I. Pipinos, Philippe Malcolm

**Affiliations:** 1 Department of Biomechanics, University of Nebraska at Omaha, Omaha, NE 68182, USA; hrazavi@unomaha.edu (H.R.); samyers@unomaha.edu (S.A.M.); 2 School of Kinesiology, University of Michigan, Ann Arbor, MI 48109, USA; 3 Department of Surgery and Research Service, Nebraska-Western Iowa Veterans Affairs Medical Center, Omaha, NE 68105, USA; ipipinos@unmc.edu; 4 Department of Surgery, University of Nebraska Medical Center, Omaha, NE 68198, USA

**Keywords:** electromyography, wearable robotics, wearable assistive device, exoskeleton, elastic exosuit, gait rehabilitation, walking assistance, arterial occlusive disease, intermittent claudication

## Abstract

**Highlights:**

**What are the main findings?**

- Elastic hip assistance can reduce biological hip moment and power in both healthy participants and patients with peripheral artery disease.
- Patients with peripheral artery disease can leverage an exosuit to help with their affected gastrocnemius.

**What is the implication of the main finding?**

- The responses to an exosuit can be different between populations, possibly because they adapt their walking to receive assistance at the most impaired joint.
- Exosuit assistance may need to be optimized specifically for each clinical population for which it is intended because the effects may be different from those in healthy individuals.

**Abstract:**

(1) Background: Peripheral artery disease (PAD) and related conditions significantly impair walking ability. Previous studies demonstrated that passive lightweight exosuits can improve walking biomechanics. However, most of these devices focus on assisting hip flexion. The aim of this study was to investigate the effects of flexion and extension assistance on joint kinetics and muscle activation. We hypothesized that there would be an optimal combination of flexion and extension assistance for measured parameters. (2) Methods: Four patients with PAD and six healthy individuals walked on a treadmill while wearing a passive exosuit with adjustable hip flexion and extension assistance. Lower limbs’ power, moment, and muscle activation were recorded. (3) Results: We found that passive hip assistance effectively reduced hip kinetics in both healthy and PAD participants. We also found different effects between the groups, with the PAD group utilizing the exosuit to reduce plantarflexion kinetics and gastrocnemius activity. (4) Conclusions: These findings suggest that patients with PAD can leverage the exosuit to ameliorate impairment-specific deficits. Future research should explore more real-world applicability of passive exosuits.

## 1. Introduction

Despite the human body’s remarkable ability to optimize walking patterns [1], individuals with impaired nervous, musculoskeletal, or vascular systems, such as patients with peripheral artery disease (PAD), often exhibit significantly compromised gait characteristics [2]. PAD is a circulatory condition in which narrowed arteries reduce blood flow to the limbs, primarily the legs. This most commonly leads to a condition called intermittent claudication, characterized by leg pain with physical activity and significantly decreased walking distances [3]. Prevalence estimates indicate that 7–12 million people in the United States and approximately 200 million people worldwide are affected by PAD [4,5,6]. The gait inefficiencies observed in patients with PAD extend beyond reduced walking distances, encompassing reduced walking speed, cadence [7], step length [8], altered ground reaction forces (GRFs) [9], and reduced lower limb joint power [10]. These biomechanical alterations contribute to a less efficient and more metabolically costly gait pattern, which can exacerbate ischemia in the legs, worsen the claudication symptoms, and further contribute to a sedentary lifestyle and reduced quality of life [11]. Additionally, patients with PAD show higher morbidity and increased cardiovascular risk at least in part due to reduced physical activity levels [12].

Simulations have suggested that assisting hip flexion could be more energy efficient than assisting other joints [13,14]. Reducing hip positive power in clinical populations can alleviate joint stress and improve energy efficiency, which is particularly beneficial for individuals with gait impairments [15,16]. The field of assistive devices has seen a significant shift from rigid exoskeletons to soft exosuits, which use lightweight, soft, and flexible materials to apply assistive forces in parallel with muscles [17,18,19]. Optimizing the assistance profile provided by these exosuits is crucial for maximizing their effectiveness [17,20]. Recent computational approaches and optimization algorithms have contributed to enhancing exosuit designs by determining optimal force profiles tailored to individual needs [21,22].

Various groups have developed passive hip exosuits that assist flexion with different torque profiles and have demonstrated the potential of these devices to improve walking efficiency [15,23,24,25,26,27]. Exosuits with passive hip flexion assistance have been shown to reduce the metabolic cost of walking by up to 4.2% in healthy older populations when compared to walking without the exosuit [25]. While several studies have investigated hip extension assistance using powered devices [28,29,30,31,32,33,34,35,36], there appears to be relatively less research on assisting hip extension with passive exoskeletons and exosuits.

Given that walking involves muscle torque generation in both hip flexion and extension, the optimal exosuit torque profile for assisting hip motion remains to be determined. Additionally, hip extension plays a crucial role in propelling the body forward, making it a key target for assistance [37]. To address this gap, we developed a passive exosuit with elastic bands that could assist both hip flexion and extension, where the force applied to each limb could be adjusted by changing the length and stiffness of the elastic bands. Therefore, this study aimed to determine how different combinations of hip flexion and extension assistance levels can be leveraged to reduce hip, knee, and ankle kinetic variables and muscle activation. We hypothesized that there is an optimal configuration for minimizing each lower limb variable.

Additionally, we aimed to compare the effects of exosuit assistance on patients with PAD to those on healthy individuals. We hypothesized that the exosuit’s effects on patients with PAD will differ from those observed in healthy populations, given that PAD substantially impacts the plantarflexor muscles and resulting gait biomechanics during push-off. Reduced plantarflexor strength and impaired push-off mechanics are key biomechanical deficits leading to functional limitations observed in PAD patients [38]. To the best of our knowledge, no prior research has investigated the effect of passive exosuits on the gait parameters of patients with PAD.

## 2. Materials and Methods

Four males and two females in the healthy group (29.7 ± 7.4 years, 70.8 ± 8.2 kg, 1.76 ± 0.08 m) and four males in the PAD group (73.5 ± 7.05 years, 78.5 ± 10.9 kg, 1.76 ± 0.03 m) participated in this study. Participants were recruited through convenience sampling. All participants provided written informed consent. The Institutional Review Board of the University of Nebraska Medical Center and the Omaha Veterans Affairs Medical Center approved this study.

Healthy participants were included if they were able to fit into the exosuit and were free of self-reported neuromotor, cardiopulmonary, or musculoskeletal disorders. Participants with PAD were included if they were able to fit into the exosuit, had a history of chronic claudication, a diagnosis of PAD, and a stable blood pressure, lipids, and diabetes regimen for >6 weeks. Exclusion criteria were the presence of rest pain or tissue loss due to PAD (Fontaine stage III and IV), acute lower extremity events secondary to thromboembolic disease or acute trauma, walking capacity significantly limited by diseases unrelated to PAD, acute injury or pain in their lower extremities, inability to follow visual cues due to blindness, or an inability to follow auditory cues due to deafness.

### 2.1. Exosuit Design

**Figure 1:**
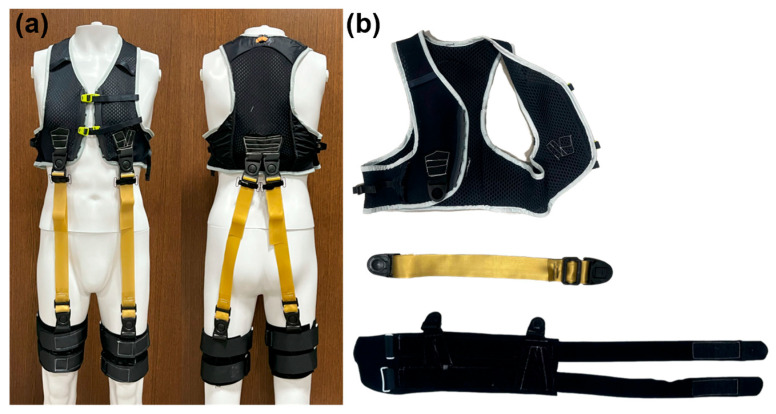
Exosuit design. (**a**) The complete exosuit assembly, comprising a garment-like torso jacket, two thigh braces, and four elastic bands, is shown. (**b**) Individual components are enlarged to highlight the texture and design. The adjustable length of the elastic bands, modified before each trial, enabled the exploration of different assistance conditions.

The exosuit had a bilateral design worn on both limbs with a garment-like texture and elastic bands (Figure 1), where the total weight of the exosuit was 0.8 kg. The elastic bands consisted of gold Thera-bands that were cut to 6.4 cm wide (~31.6 N resistance at 100% elongation). The device consisted of a torso jacket connected to thigh braces. The front band stretched and stored energy when the hip extended and then shortened in parallel with the hip flexors when the leg moved forward. Similarly, the dorsal band assisted hip extension after heel strike and stored energy when the leg moved forward. To investigate various torque conditions, the length of the elastic bands was adjusted prior to each trial.

### 2.2. Protocol

We employed a within-subjects design where each participant walked for one minute in each of nine different conditions in a randomized order on a treadmill (Bertec, Columbus, OH, USA) at a speed of 0.8 m∙s^−1^. One PAD participant walked at a speed of 0.5 m·s⁻^1^ because he could not maintain 0.8 m∙s^−1^. The first author ran a program to generate a randomized sequence of walking conditions prior to each data collection session. After each walking condition, participants were allowed to rest as long as they requested. The conditions and exosuit setup involved adjusting the bands’ lengths and hence the force applied by the exosuit on each leg prior to each condition. Participants stood in a double support position facing forward, and we measured the slack length of the elastic bands when they wore the exosuit. “High” was 1/3 of the slack length (maximum force applied), “mid” was 2/3 of the slack length (medium force applied), and “no” meant that the bands were longer than the slack length (such that no force was applied during any part of the gait cycle). Each condition was a combination of high, mid, and no assist in the front and back, thus creating a wide range of forces applied on both legs equally by the exosuit. PAD participants came to the laboratory for two sessions. The first session was focused on consent and walking with the exosuit with different band tensions for a total of nine minutes. Healthy participants required only a single session to complete all activities. The data collections happened at the Biomechanics Research Building at the University of Nebraska at Omaha. Neither the participants nor the researchers were blinded to the intervention. However, the participants were not informed of any aim or hypothesis.

### 2.3. Measurements

We recorded the activity of the primary flexors and extensors of the leg (soleus, gastrocnemius medialis, tibialis anterior, vastus medialis, rectus femoris, and biceps femoris) using Delsys Trigno Avanti Surface EMG sensors (Delsys Inc., Natick, MA, USA). The EMG signals were sampled at 2000 Hz. We followed SENIAM guidelines for the placement of the electrodes after cleaning the skin surface with alcohol [39]. We measured GRFs using a force treadmill (Bertec, Columbus, OH, USA), which was calibrated with an instrumented pole (C-motion, Germantown, MD, USA) [40] and had an accuracy of ±5 mm for the center of pressure. Three-dimensional lower limb kinematic data were captured using a sixteen-camera motion capture system (VICON Vero, Oxford Metrics, Yarnton, UK; 2000 Hz) from 26 reflective markers placed on lower limb anatomical landmarks according to a modified Helen Hayes marker set [41]. The motion capture system was calibrated using an LED wand. Calibrations were repeated until the world error reached below 0.2 mm. The forces exerted on the right leg by elastic bands during the trial were measured using two load cells (Futek, Irvine, CA, USA; 1000 Hz, measurement range 1000 lbs) connected to a commercially available actuation system (HuMoTech, Pittsburgh, PA, USA) mounted between the torso jacket and elastic bands. To ensure consistency, markers and EMG sensors were always placed by the same researcher.

### 2.4. Data Processing

**Figure 2:**
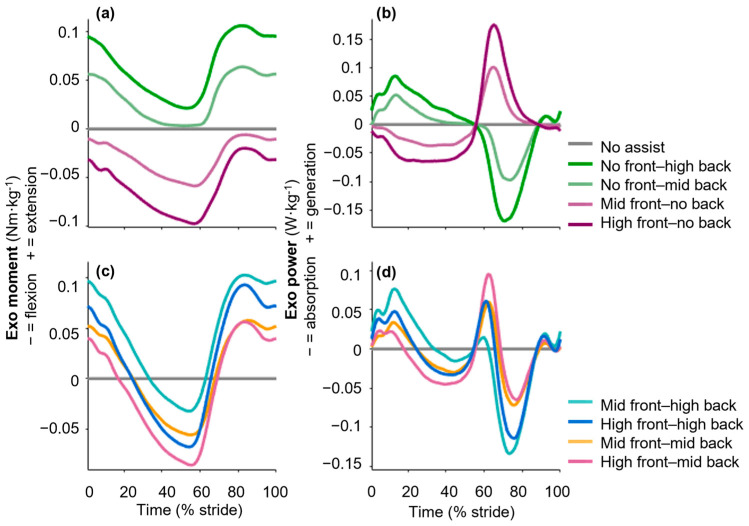
Exosuit sagittal plane moment and power in both healthy and PAD groups. Different band length conditions apply a range of exosuit (**a**,**c**) moments and (**b**,**d**) powers. Figures (**a**,**b**) show exosuit (**a**) moments and (**b**) powers under conditions with either only flexion (purple) or only extension (green) assistance. Figures (**c**,**d**) depict exosuit (**a**) moments and (**b**) powers for conditions with a combination of flexion and extension assistance. Additionally, exosuit moments and powers with standard errors for each group are presented in Appendix A (Figure A2 and Figure A3). Plots for a representative participant, including inter-stride standard deviations, are shown in Figure A1.

We performed inverse dynamics analyses on the motion capture and force platform data to calculate joint moments and powers. We calculated the net moments of the exosuit by multiplying each load cell measurement by the moment arm (10 cm, perpendicular distance between the bands and hip joint, assuming an average-sized person) [42] (Figure 2). We calculated the power delivered by the exosuit by multiplying the moment by the angular velocity from motion capture. The biological moments and powers were calculated by subtracting the exosuit contribution from the external hip joints and moments. The GRF and load cell signals were smoothed with a 10 Hz low-pass filter, and the surface EMG data were band-pass filtered (50–400 Hz) and rectified. A moving root mean square with a window of 100 ms was then applied, and all EMG values were normalized to the value of the no-assist condition so that the no-assist condition represented a value of 100.

### 2.5. Statistical Analysis

We used mixed-effects model analyses with participants as random factors to determine the effects of the exosuit’s extension and flexion peak torques on hip, knee, and ankle moments, powers, and muscle activation [43]. We included the first-order terms of the assistive torques as well as the interaction term, consistent with previous studies that analyzed effects on biomechanics and muscle activation in ankle exoskeletons using linear models with spring stiffness as an independent parameter. For each biomechanical parameter, we evaluated the following relationship:

Outcome ~ c_1∙_τ_dors_ + c_2∙_τ_front_ + c_3∙_τ_dors∙_τ_front_ + c_4_ (1)

where τ_dors_ is the peak torque of the dorsal elastic bands that assist hip extension, τ_front_ is the peak torque of the frontal elastic bands that assist hip flexion, c_1–3_ are coefficients, and c_4_ is a constant term. To avoid overfitting, terms that did not significantly contribute were removed using a stepwise elimination procedure whereby the least significant terms were removed until only significant terms remained [44,45]. This approach of investigating the effects of two parameters and their interaction, and modifying the used terms using backward elimination, is similar to other biomechanics studies [46]. We used a paired *t*-test with the Holm–Šídák correction for multiple comparisons [47] to compare each condition with the no-assist condition. The greatest reductions and increases were determined by identifying the largest differences between the no-assist condition and all other conditions, calculated separately for the positive and negative portions of each variable. When statistically significant, we reported the greatest reduction (increase) for each variable based on the following equation:

Reduction (increase) (%) = (Condition with greatest reduction (increase) − No assist condition) ÷ No assist condition × 100%(2)

## 3. Results

### 3.1. Increasing Hip Assistance Reduced Biological Hip Kinetics

**Figure 3:**
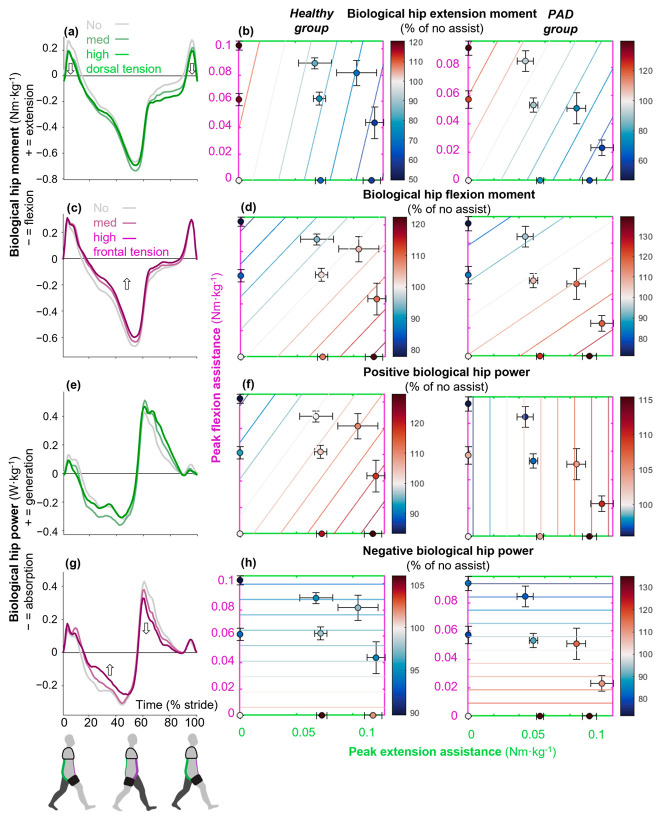
Effects on sagittal plane biological hip kinetics. Time-series plots show the effects of different levels of only flexion assistance (purple) or only extension assistance (green) from the exosuit on the biological (**a**,**c**) hip moment and (**e**,**g**) power. Subplot (**b**) represents the effects on only the positive portion of the biological hip moment (extension moment). Subplot (**d**) represents the effects on the negative portion of the biological hip moment (flexion moment). Dots represent the means of each condition expressed as a percentage of the absolute value of the no-assist condition. Standard error bars around each dot represent the participants’ variabilities. Contour lines show the significant effects of combinations of different levels of flexion assistance on the vertical axis and the different levels of extension assistance on the horizontal axis. When the contours do not show effects along one axis, those effects are insignificant. In summary, the contours could be used to estimate how to best target a specific variable; for example, (**b**) shows that the biological hip extension moment in healthy young adults is reduced by increasing the tension of the dorsal bands and reducing the tension of the frontal bands. Subplots (**d**–**h**) show (**d**) biological hip flexion moment and (**f**) positive and (**h**) negative hip powers. The left column shows results in healthy young adults, and the right column shows results in patients with PAD.

Maximizing hip extension assistance and providing no hip flexion assistance (which would otherwise resist hip extension) minimized the biological hip extension moment at early stance and late swing in the healthy and PAD groups (*p*_τdors_ ≤ 0.01 in the healthy and PAD group; mixed-effects model where *p*_τdors_ is the *p*-values of the effects of the dorsal bands that assist extension; Figure 3a,b). The greatest reductions were 40.2 ± 14.8% and 40.1 ± 14.7% in the healthy and PAD groups (*p* ≤ 0.01 and 0.05; Holm–Šídák-corrected paired *t*-tests versus no-assist; Table A1). The greatest reduction occurred in both cases with maximal extension assistance (0.12 and 0.10 Nm·kg^−1^). In the healthy group, the greatest reduction occurred with zero hip flexion assistance; in the PAD group, it occurred with medium hip flexion assistance (0.02 Nm·kg^−1^; Table 1). On the other hand, maximizing hip flexion assistance and minimizing hip extension assistance increased biological hip extension moment in both groups (*p*_τfront_ ≤ 0.05 and *p*_τfront_ ≤ 0.01 in the healthy and PAD group; mixed-effects model where *p*_τfront_ is the *p*-values of the effects of the frontal bands that assist flexion; Figure 3a,b). The greatest increases were 20.7 ± 28% and 40 ± 23% in the healthy and PAD groups and occurred in conditions with maximum flexion assistance (0.1 and 0.09 Nm·kg^−1^) and no extension assistance (Table 1). However, we did not see the effect of hip extension assistance in the EMG results. Although increasing extension assistance did not reduce biceps femoris EMG in the healthy or PAD group (*p*_τdors_ ≥ 0.05; Table A1), increasing flexion assistance increased biceps femoris EMG in both groups (*p*_τfront_ ≤ 0.01). It is possible that there could be an effect on the gluteus EMG. However, we did not measure this muscle in this study.

Maximizing hip flexion assistance and providing no hip extension minimized biological hip flexion moment around mid-stance in the healthy and PAD groups (*p*_τfront_ ≤ 0.01; Figure 3d). The greatest reductions in biological hip flexion moment were 22.2 ± 15.5% and 28.6 ± 5.2% in the healthy and PAD groups (*p* ≤ 0.01 in healthy; Holm–Šídák corrected paired-t tests versus no-assist; Table A1). In both groups, the highest reduction occurred with maximal hip flexion assistance (0.10 and 0.09 Nm·kg^−1^) and zero hip extension assistance (Table 1). On the other hand, maximizing hip extension assistance and providing no flexion assistance increased biological hip flexion moment in both groups (*p*_τdors_ ≤ 0.01; Figure 3d). The highest increases were 21.6 ± 24% and 40 ± 36% in the healthy and PAD groups and occurred in a condition with maximum extension assistance (0.12 and 0.09 Nm·kg^−1^) and no flexion assistance (Table 1). Although the effect of flexion assistance did not reflect in rectus femoris EMG in any groups, the effect of extension assistance was observed only in the PAD group (*p*_τdors_ ≤ 0.01; Table A1). It is possible that hip flexion assistance reduces activation in deeper muscles that could not be measured, such as the major psoas.

Maximizing hip flexion assistance and providing no hip extension minimized positive biological hip power during the swing but only in the healthy group (*p*_τfront_ ≤ 0.01; Figure 3f). On the other hand, maximizing hip extension assistance and providing no flexion assistance (*p*_τdors_ ≤ 0.01; Figure 3d) increased positive biological hip power in both groups. The greatest increases were 29.8 ± 14.3% and 15.5 ± 18.6% in the healthy and PAD groups, and they occurred in a condition with maximum extension assistance (0.12 and 0.09 Nm·kg^−1^) and zero flexion assistance (Table 1).

Maximizing hip flexion assistance minimized negative biological hip power during mid-stance, both in the healthy and PAD groups (*p*_τfront_ ≤ 0.05 and ≤ 0.01; Figure 3h). The greatest reductions were 10.3 ± 20.8% and 20.9 ± 19.4% (Table A1). In both groups, the greatest reduction occurred with zero hip extension assistance. In the healthy group, the greatest reduction occurred with maximal hip flexion assistance (0.10 Nm·kg^−1^); in the PAD group, it occurred with medium hip flexion assistance (0.06 Nm·kg^−1^). However, extension assistance did not have any significant effects on negative biological hip power (*p*_τdors_ ≥ 0.01; Table A1).

### 3.2. Increasing Hip Flexion Assistance Reduced Plantarflexion Moments

**Figure 4:**
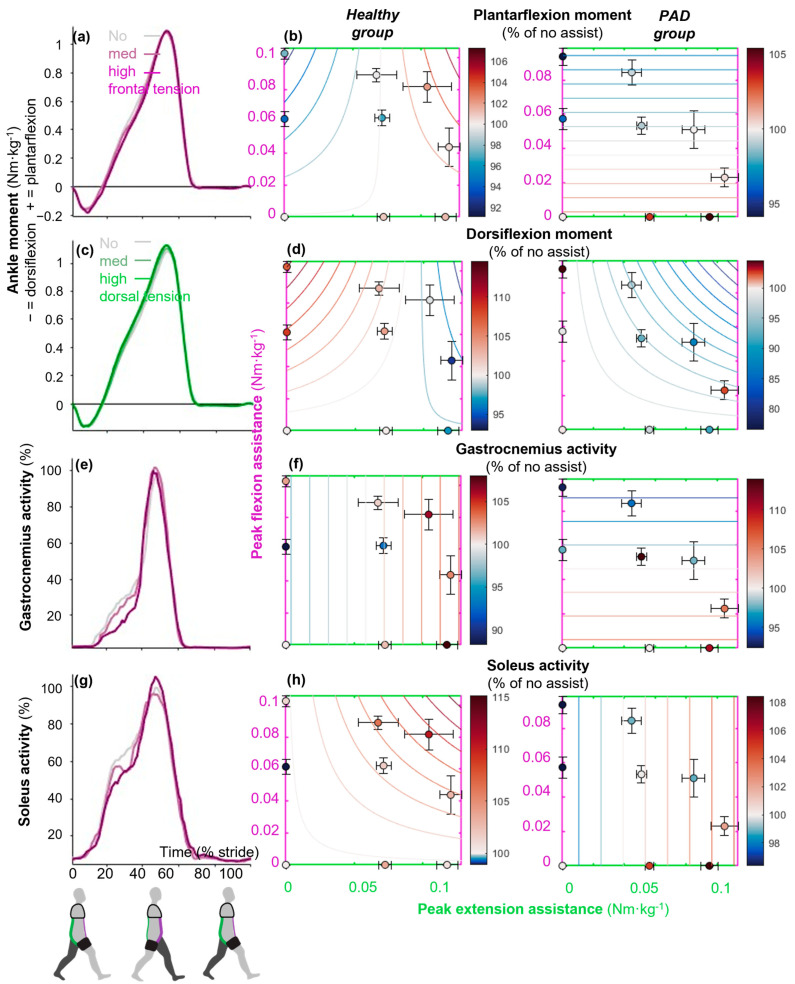
Effects on sagittal plane ankle kinetics. Time-series plots show the effects of different levels of only flexion assistance (purple) or only extension assistance (green) from the exosuit on the (**a**,**c**) ankle moment, (**e**) gastrocnemius medialis, and (**g**) soleus activity in the PAD group. Dots represent the means of each condition expressed as a percentage of the absolute value of the no-assist condition. Standard error bars around each dot represent the participants’ variabilities. Contour plots show the effects of combinations of different levels of flexion assistance on the vertical axis and different levels of extension assistance on the horizontal axis. For example, (**b**) shows that plantarflexion moment in the PAD group is reduced by increasing frontal band tensions and that dorsal band tension has no effect. Subplots (**d**–**h**) show (**d**) dorsiflexion moment, (**f**) gastrocnemius activity, and (**h**) soleus activity. The left column shows results in healthy young adults, and the right column shows results in PAD patients.

Maximizing hip flexion assistance minimized plantarflexion moment around mid-stance in the healthy and PAD groups (*p*_τfront_ ≤ 0.05 and ≤0.01; Figure 4a,b). The greatest reductions were 5.6 ± 12.2% and 5.9 ± 5.8%, respectively, which occurred in the conditions with medium (0.06 Nm·kg^−1^) and maximum (0.09 Nm·kg^−1^) hip flexion assistance and zero extension assistance. There were no significant effects of extension assistance on plantarflexion moment in either group (*p*_τdors_ ≥ 0.01; Table A1). Maximizing hip flexion assistance also minimized gastrocnemius activity in the PAD group around mid-stance (*p*_τfront_ ≤ 0.05; Figure 4e,f). The greatest reduction was 7.15 ± 5.8%, which occurred in the condition with maximum flexion assistance (0.09 Nm·kg^−1^) and zero extension assistance (Table 1). Although there was no significant effect of hip flexion assistance on gastrocnemius activity in the healthy group, maximizing extension assistance increased this muscle’s activity (*p*_τdors_ ≤ 0.05). However, these effects were not reflected in soleus activity or positive ankle power, as increasing flexion or extension assistance did not reduce either measure.

There was an interaction effect between flexion and extension assistance on dorsiflexion moment in both groups (*p*_τdors∙τfront_ ≤ 0.01 and ≤ 0.01 in the healthy and PAD groups). The greatest reductions were 5.8 ± 11.5% and 12.4 ± 18.8% in each group. These greatest reductions occurred in conditions with medium (0.04 Nm·kg^−1^) and maximum (0.05 Nm·kg^−1^) flexion and maximum extension assistance (0.12 and 0.08 Nm·kg^−1^) in the healthy and PAD groups, respectively (Table 1). The greatest increases in negative ankle moment were 8.5 ± 21% and 4.6 ± 14.3% and occurred with medium (0.06 Nm·kg^−1^) and maximum (0.09 Nm·kg^−1^) flexion assistance and zero extension assistance in the healthy and PAD groups, respectively (Table 1). However, the effects on tibialis anterior activity were not significant.

### 3.3. Maximizing Hip Extension Assistance Reduced Knee Power

**Figure 5:**
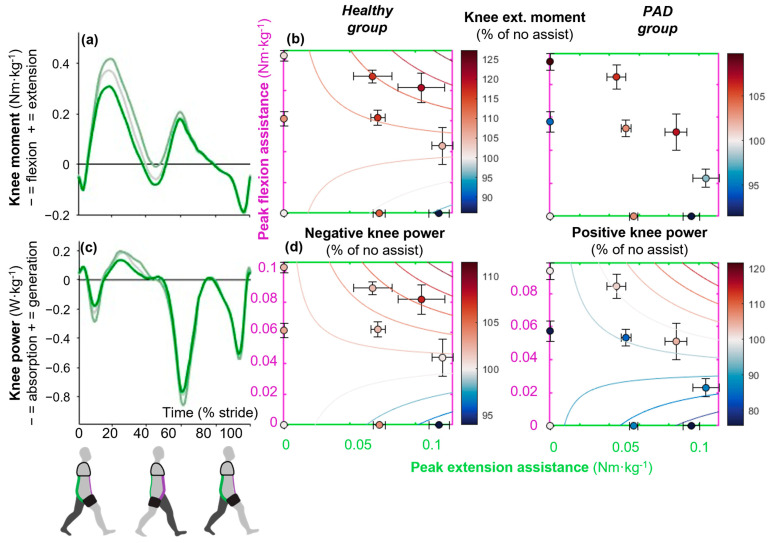
Effects on sagittal plane knee kinetics. Time-series plots show the effects of different levels of only extension assistance (green) from the exosuit on the knee (**a**) moment and (**c**) power. Dots represent the means of each condition expressed as a percentage of the absolute value of the no-assist condition. Standard error bars around each dot represent the participants’ variabilities. Contour plots show the effects of combinations of different levels of flexion assistance on the vertical axis and different levels of extension assistance on the horizontal axis on (**b**) knee extension moment and (**d**) knee power. For example, subplot (**b**) shows that the knee extension moment in healthy young adults is reduced by increasing dorsal band tension and reducing frontal band tension. The left column shows results in healthy young adults, and the right column shows results in PAD patients.

Maximizing hip extension assistance and providing no flexion assistance minimized knee extension moment around mid-stance but only in the healthy group (*p*_τdors_ ≤ 0.05, *p*_τdors∙τfront_ ≤ 0.01; Figure 5a,b). The greatest reduction was 14.1 ± 17.6%, which occurred in the condition with maximum extension assistance (0.12 Nm·kg^−1^) and zero flexion assistance (Table 1). On the other hand, the greatest increase was 20 ± 8.7% and happened in the condition with maximum flexion (0.08 Nm·kg^−1^) and extension (0.1 Nm·kg^−1^) assistance (Table 1). However, the effects on vastus medialis or rectus femoris did not support this trend.

Maximizing hip extension assistance and providing no flexion assistance minimized negative and positive knee power in the healthy and PAD groups, respectively (*p*_τdors_ ≤ 0.05, *p*_τdors∙τfront_ ≤ 0.01 in both; Figure 5c,d). In the healthy group, the greatest reduction in negative knee power was 6.0 ± 5.9% in the condition with maximum extension assistance (0.12 Nm·kg^−1^) and zero flexion assistance (Table 1). The greatest increase was 7.6 ± 8.2, which occurred in the condition with maximum flexion (0.08 Nm·kg^−1^) and extension (0.1 Nm·kg^−1^) assistance (Table 1). In the PAD group, the greatest reduction in positive knee power was 24.3 ± 9.3%, which occurred in the condition with maximum extension assistance (0.09 Nm·kg^−1^) and zero flexion assistance (Table 1). The greatest increase was 4.2 ± 19.5%, which occurred in a condition with maximum flexion (0.05 Nm·kg^−1^) and extension (0.08 Nm·kg^−1^) assistance (Table 1).

## 4. Discussion

This study aimed to evaluate the effects of different levels of hip flexion and extension assistance using a passive exosuit on hip, knee, and ankle muscle activity, joint moments, and powers during walking in patients with PAD and healthy individuals. We hypothesized that there is an optimal combination of flexion and extension assistance for minimizing each variable. We also hypothesized that the exosuit’s effects on patients with PAD differ from those observed in healthy populations, given that PAD substantially impacts the plantarflexor muscles and resulting gait biomechanics during push-off. The results partially supported these hypotheses. We found that conditions that maximized hip extension or flexion assistance minimized biological hip extension and flexion moment, respectively, in both groups. We discovered that maximizing hip flexion assistance reduced negative biological hip power in both groups. Minor differences between healthy and PAD participants were observed. The exosuit’s flexion assistance reduced gastrocnemius EMG only in patients with PAD, while it reduced positive biological hip power only in healthy participants.

Our exosuit’s weight (0.8 kg) was slightly more than that of some previous exosuit designs [25,48] (0.63 and 0.65 kg) but remained lighter than other passive exosuits, such as 2.8 [49], 1.9 [50], 1.8 [51], and 1.3 [52] kg in other studies. Our exosuit applied a peak flexion torque of 0.12 Nm·kg^−1^, which exceeded the maximum torque previously applied by passive hip exosuits (0.05 and 0.02 Nm·kg^−1^ [25,48]). Although this torque was slightly lower than the optimum flexion torque magnitude (0.15 Nm·kg^−1^) identified by another study for reducing metabolic cost [15], it is important to note that their optimal profile was determined using a tethered exosuit providing only flexion assistance with no extension torque and was highly individualized through human-in-the-loop optimization. Moreover, their assistance profile differed in both magnitude and timing from ours, making their reported optimum specific to their setup and control strategy.

While many studies have shown that passive hip assistive devices can reduce the metabolic cost of walking [25,48,50,51], fewer studies have examined their effects on joint kinetics and muscle activation [23,26]. Passive hip flexion devices, when applying low, consistent torque during mid-stance, have reduced energy cost by 3–4% in older adults without altering spatiotemporal parameters [25,48]. Other designs, including biarticular springs, have demonstrated further metabolic cost reductions up to 7% by coordinating assistance across joints [50]. While these findings highlight the energy-saving potential of passive devices, most studies have not assessed joint-level biomechanics.

Our finding that maximizing hip extension or flexion assistance minimized biological hip extension and flexion moments is aligned with the results of Kim et al., who found similar results with two exosuits that provided hip extension and hip flexion assistance [15,53]. The achieved reductions in biological hip flexion moment (0.06 and 0.04 Nm·kg^−1^ in the healthy and PAD groups, respectively) by maximizing hip flexion assistance are close to those reported in the previous literature, which found a 0.09 Nm·kg^−1^ reduction in healthy young participants [15]. However, their exosuit was powered, and ours was passive. The finding of reducing biological hip power by maximizing hip flexion assistance is consistent with that of other studies that found a reduction in positive [23] and negative biological hip power [15] using hip flexion assistance. We found that maximizing hip flexion assistance minimized plantarflexion moment in both groups and gastrocnemius activity in the PAD group. This is similar to trends showing reduced plantarflexion moment [15] and gastrocnemius EMG [23] in other studies. However, these reductions were found in healthy participants. Our finding of minimized knee extension moment when maximizing hip extension assistance in healthy individuals is again supported by the results of Kim et al. for a powered hip extension exosuit [53].

**Figure A4:**
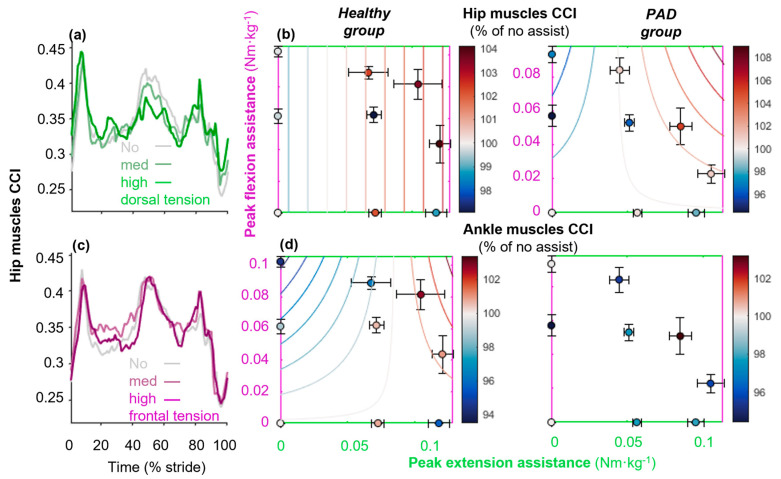
Co-contraction index (CCI) for hip and ankle muscles. Figures (**a**,**c**) depict the hip muscle CCI during the stride cycle. For hip muscles, we considered the rectus femoris and biceps femoris since we did not measure deeper muscles. Effects of exosuit torque profiles on (**b**) hip and (**d**) ankle muscle CCI for the healthy and PAD groups are shown.

Contrary to expectations, the reductions in kinetics were not always associated with the reductions in the associated muscle activation patterns. To assess these cases, we investigated the effects on muscle co-contraction. We calculated the mean co-contraction index (CCI) across the full gait cycle using normalized EMG from the hip (biceps femoris, rectus femoris), ankle (tibialis anterior, medial gastrocnemius), and knee (vastus medialis, biceps femoris) muscles. At each time point, we computed the CCI as the ratio of the lower EMG signal to the sum of both signals and then averaged across the gait cycle for each condition and subject [54]. For hip muscles, increasing hip extension assistance led to increased CCI in both groups (healthy: Coef_τdors_ = 35 ± 17, p_τdors_ ≤ 0.05; PAD: Coef_τdors_ = −54 ± 20, p_τdors_ ≤ 0.05, Coef_τdors∙τfront_ = 1308 ± 344, p_τdors∙τfront_ ≤ 0.01; Figure A4b). However, for ankle muscles, we observed the same effects only in the healthy group (Coef_τdors_ = −63 ± 30.5, p_τdors_ ≤ 0.05, Coef_τdors∙τfront_ = 729 ± 330, p_τdors∙τfront_ ≤ 0.01; Figure A4d). No significant effect was observed for knee muscles in either group. Although increasing hip extension assistance reduced the biological hip extension moment in both groups, no similar reduction was observed in biceps femoris activity. This may be due to an increase in CCI between the biceps femoris and rectus femoris with increased hip extension assistance. Additionally, while increasing hip extension assistance increased gastrocnemius activity in the healthy group, no such effects were seen in the PAD group. This could be explained by the increase in CCI between the gastrocnemius and tibialis anterior in the healthy group, which was not observed in the PAD group. Another possible explanation is that the study did not measure all muscles involved (such as gluteus maximus and iliopsoas activity), which could account for some of the observed kinetic changes. In addition, the central nervous system may be adapting to the exosuit assistance by modulating muscle activation patterns in ways that are not directly reflected in reductions in the measured EMG of specific muscles [55]. Furthermore, the exosuit assistance may be redistributing loads across multiple muscle groups, leading to subtle changes in activation patterns that are not captured by measuring individual muscles [56].

Our findings suggest that targeted assistance at the hip can influence distal muscle activation patterns, which are consistent with the soleus–hip trade-off hypothesis [57]. The decrease in gastrocnemius activity in response to the exosuit’s assistance aligns with the hypothesis that enhanced ankle push-off can reduce hip muscle moments, suggesting a shift in the biomechanical burden from the hip to the ankle and vice versa. This is also consistent with another study [15], in which decreasing ankle plantarflexion moment suggests that they rely more on the hip and less on the ankle.

The observed difference between the PAD and healthy group in leveraging the exosuit to assist their ankle may reflect a strategic adaptation by individuals with PAD to address their most problematic joints. Individuals with PAD demonstrate impaired ankle joint mechanics, characterized by reduced plantarflexor power and altered muscle control strategies, even before the onset of claudication pain [58]. When provided with exosuit assistance, patients with PAD appear to leverage this support more effectively at the ankle, possibly as a compensatory mechanism for their inherent vascular insufficiency and associated muscular weakness and control deficits [59]. In contrast, healthy individuals may not require or benefit as much from ankle assistance as their vascular and neuromuscular function is intact. This differential response to exoskeleton assistance aligns with the principle of targeted intervention, where assistive devices are most effective when addressing specific functional limitations [60].

A key limitation of our study is the absence of metabolic cost measurements, which prevents us from determining whether the observed reductions in joint moments and muscle activation translate into actual improvements in walking efficiency. Another limitation of this study is that only participants with PAD received a habituation period, while healthy participants did not. Although not designating a habituation period to passive exosuits is consistent with previous studies [23,51], this might have influenced how each group responded to the exosuit. Furthermore, healthy participants were probably walking slower than their preferred speed [61], which may limit the comparability of results between groups. Additionally, the sample size was relatively small, with only six healthy participants and four PAD participants, which may limit the generalizability of the study findings. However, we performed a post-hoc statistical power analysis of the linear mixed-effects model coefficients using a simulation approach [62,63,64], which involved generating simulated data based on the model coefficients and re-running the simulations 1000 times. In most cases, the statistical power was greater than 0.80, suggesting that the sample size was sufficient to detect the observed effects.

Additionally, the assumed 10 cm moment arm, based on typical anatomical dimensions [42], may limit the accuracy of moment measurements and control. Future studies should use direct measurements, such as sensors or markers, to improve biomechanical precision by estimating individual differences in moment arms and tracking how these moment arms change throughout the gait cycle. This study focused on the biomechanical effects of the exosuit, without optimizing its configuration or material design. Future work could explore how factors such as the band height of the trunk and thigh affect moment arm changes and estimate the optimal configuration through predictive simulations. Additionally, investigating alternative materials for the non-elastic components could enhance rigidity. Moreover, the assumption of symmetry between the right and left elastic bands may have introduced some bias. Due to limitations in instrumentation and the desire to minimize added weight, we only added load cells on the right side and tried to match the tension levels between the sides. Minor inconsistencies in the tension from the left side could have influenced the kinetics and EMGs of the right side.

Notably, the PAD group was, on average, 10% heavier than the healthy group. In this study, we applied the same exosuit torque profiles to both groups by shortening an elastic band over similar percentages of its length, resulting in similar force levels regardless of body weight. In cases where smaller reductions were observed in the PAD group, such as in knee negative power, this may be due to the fact that the applied force was lower relative to body weight. However, this effect was only significant for knee power in the healthy group, suggesting that the lack of force scaling did not significantly impact the results. Future studies should consider normalizing force levels to body weight to improve comparability between groups. Additionally, previous research has shown that metabolic adaptations to exosuits can occur over multiple training sessions, and the current study design did not capture any longer-term effects [65]. Toward these goals, our research team is currently performing research with a further-developed version of the exosuit in the home setting of a rural patient population. New methods, such as open-source marker-less motion capture [66], allow us to record the effects of these exosuits in more real-world settings compared to short-term treadmill walking. This is important because walking during daily life is often very different from steady-state treadmill walking [67]. Finally, the study focused on steady-state treadmill walking at a single speed, which may not fully represent the diverse walking conditions encountered in daily life, particularly for individuals with PAD. Adapting exoskeleton assistance to real-life situations remains a significant challenge despite laboratory studies showing benefits like reduced metabolic cost and improved walking performance. Most research still focuses on steady-state walking in controlled settings, not fully capturing the variability of real-world movement. Future studies need to explore how exosuits can adapt to different terrains, speeds, and tasks for better applicability in everyday life. Future work will also need to evaluate whether the observed reductions in joint kinetics and EMGs translate to functional benefits. Similar to other studies with new devices [15,48], this study focused on short walking bouts in order to investigate feasibility and initial effects while minimizing risks for the clinical population. Future research will have to evaluate effects on clinically relevant functional tests such as the 6-min walking test, metabolic cost of transport, quality-of-life surveys, etc.

## 5. Conclusions

In conclusion, our findings show that increasing hip assistance can both reduce and increase biological hip kinetics depending on the type of assistance applied. Hip extension assistance tended to reduce hip extension moments by up to 40%, while hip flexion assistance reduced hip flexion moments by up to 28%; however, in some cases, assistance led to increased joint kinetics. These effects varied between healthy individuals and patients with PAD, especially in the ankle. Notably, PAD participants appeared to leverage the exosuit more effectively to support ankle function, suggesting its potential as a targeted intervention. The interplay between flexion and extension assistance is complex and not fully resolved in our results. We believe this highlights the need for personalized exosuit tuning based on the specific gait impairments of the user. While our study demonstrates the potential of passive hip assistance in modulating joint kinetics and muscle activity, future research should address long-term adaptations, real-world applicability, and the development of more comprehensive assessment methods to fully elucidate the vascular and neuromuscular responses to exosuit assistance. As exosuit technology continues to advance, it holds significant promise for enhancing mobility and independence in individuals with PAD and other walking limitations.

## Figures and Tables

**Table 1:** Summary table of conditions that produced maximum reduction and increase in variables with significant effects, as determined by mixed-effects model analysis (detailed in Table A1).

Variables	Group	Tension Conditions for Maximum Reduction	Tension Conditions for Maximum Increase
Positive biological hip moment (Nm∙kg^−1^)	Healthy	No front-high back	High front-no back
	PAD	Mid front-high back	High front-no back
Negative biological hip moment (Nm∙kg^−1^)	Healthy	High front-no back	No front-high back
	PAD	High front-no back	No front-high back
Positive biological hip power (W·kg^−1^)	Healthy	High front-no back	No front-high back
	PAD	High front-no back	No front-high back
Negative biological hip power (W·kg^−1^)	Healthy	High front-no back	No front-mid back
	PAD	Mid front-no back	No front-high back
Mean biceps femoris activity (%)	Healthy	No front-high back	High front-no back
	PAD	Mid front-high back	High front-no back
Mean rectus femoris activity (%)	PAD	High front-no back	Mid front-high back
Positive ankle moment (Nm∙kg^−1^)	Healthy	Mid front-no back	High front-high back
	PAD	High front-no back	No front-high back
Negative ankle moment (Nm∙kg^−1^)	Healthy	Mid front-high back	Mid front-no back
	PAD	High front-high back	High front-no back
Positive ankle power (W·kg^−1^)	Healthy	No front-mid back	High front-mid back
	PAD	Mid front-no back	Mid front-high back
Negative ankle power (W·kg^−1^)	Healthy	High front-no back	No front-high back
Mean soleus activity (%)	Healthy	Mid front-no back	High front-high back
	PAD	Mid front-no back	No front-high back
Mean gastrocnemius medialis activity (%)	Healthy	Mid front-no back	No front-high back
	PAD	High front-no back	Mid front-mid back
Positive knee moment (Nm∙kg^−1^)	Healthy	No front-high back	High front-high back
Positive knee power (W.kg^−1^)	PAD	No front-high back	High front-high back
Negative knee power (W·kg^−1^)	Healthy	No front-high back	High front-high back
Mean vastus medialis activity (%)	PAD	No front-high back	High front-no back

**Figure A1:**
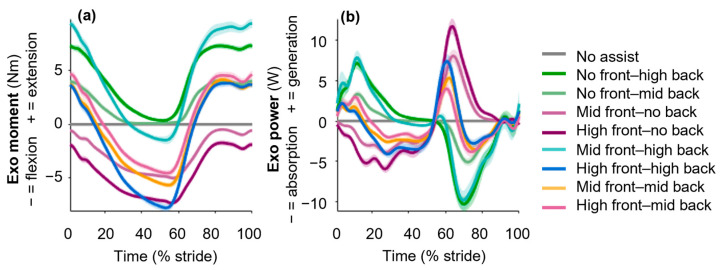
Exosuit sagittal plane moment and power (not normalized to body mass) in a representative healthy subject. Different band length conditions apply a range of exosuit (**a**) moments and (**b**) powers. The shaded area represents the inter-stride standard deviation.

**Figure A2:**
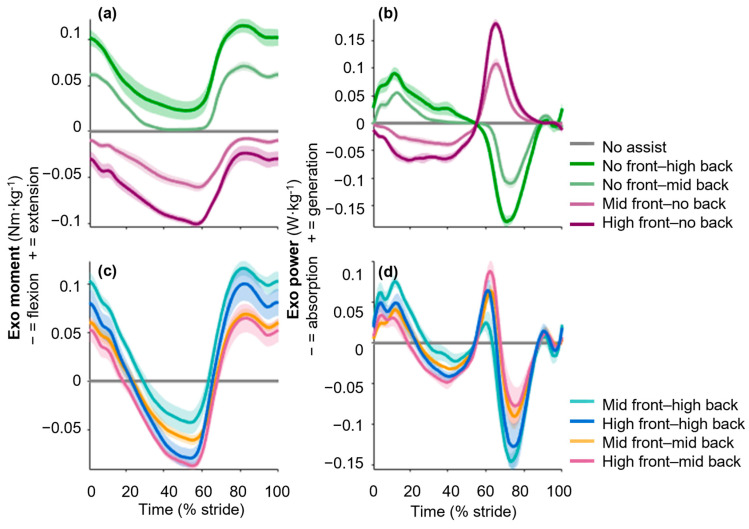
Exosuit sagittal plane moment and power in the healthy group. Different band length conditions apply a range of exosuit (**a**,**c**) moments and (**b**,**d**) powers. The shaded area represents standard errors.

**Figure A3:**
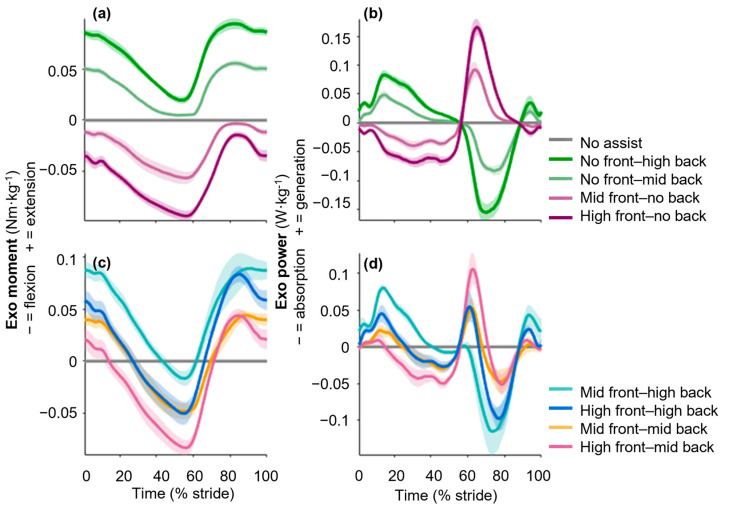
Exosuit sagittal plane moment and power in the PAD group. Different band length conditions apply a range of exosuit (**a**,**c**) moments and (**b**,**d**) powers. The shaded area represents the standard error.

## Data Availability

The data presented in this study are publicly available in Supplementary Data File S1.

## Supplementary Materials

The following supporting information can be downloaded at: https://www.mdpi.com/article/10.3390/s25113368/s1, Data File S1: MATLAB 2023b (MathWorks Inc., Natick, MA, USA) source data with biomechanical time-series and derived scalars.

## Abbreviations

The following abbreviations are used in this manuscript:

PAD	peripheral artery disease
EMG	electromyography
GRF	ground reaction force

## Appendix A

**Table A1:** Effects of exosuit peak moments on biomechanical variables. Rows contain the analyzed biomechanical variables. Columns contain the coefficients of the significant terms of the mixed-effects model as well as the percent change in the condition with the greatest reduction and the condition with the greatest increase and the peak moments at which this occurred. ** represents *p* < 0.01, and * represents *p* < 0.05.

Variable	Group	Coefficients				Maximum Reduction			Maximum Increase		
		τ_dors_	τ_front_	τ_dors_∙τ_front_	Constant	% (SD)	τ_dors_ (Nm·kg^−1^)	τ_front_ (Nm·kg^−1^)	% (SD)	τ_dors_ (Nm·kg^−1^)	τ_front_ (Nm·kg^−1^)
Positive biological hip moment (Nm∙kg^−1^)	Healthy	−436 **	120 *	ns	105	40.2 (14.8) **	0.12	0	20.7 (28)	0	0.1
	PAD	−490 **	291 **	ns	102	40.1 (14.7) *	0.10	0.02	40 (23)	0	0.09
Negative biological hip moment (Nm∙kg^−1^)	Healthy	180 **	−201 **	ns	99	22.2 (15.5) **	0	0.10	21.6 (24) *	0.12	0
	PAD	237 **	−389 **	ns	110	28.6 (5.2)	0	0.09	40 (36)	0.09	0
Positive biological hip power (W·kg^−1^)	Healthy	210 **	−180 **	ns	102	16.7 (22)	0	0.10	29.8 (14.3) **	0.12	0
	PAD	150.8 **	ns	ns	95	4.5 (10.2)	0	0.09	15.5 (18.6)	0.09	0
Negative biological hip power (W·kg^−1^)	Healthy	ns	−85.5 *	ns	101.5	10.3 (20.8)	0	0.10	6.18 (14.5)	0.07	0
	PAD	ns	−534 **	ns	125	20.9 (19.4)	0	0.06	35.1 (63)	0.09	0
Mean biceps femoris activity (%)	Healthy	ns	147 **	ns	94	8.9 (9.2)	0.08	0	13.5 (11.5)	0	0.1
	PAD	ns	197 **	−2367 *	98	9.3 (14)	0.08	0.05	14 (7.7)	0	0.09
Mean rectus femoris activity (%)	Healthy	ns	74.7 *	ns	102	0	0	0	13.8 (17.8)	0.1	0.08
	PAD	279 **	ns	ns	96	2.4 (2.7)	0	0.09	30.2 (30.8)	0.1	0.02
Positive ankle moment (Nm∙kg^−1^)	Healthy	ns	−85.2 *	1191 **	100	5.6 (12.2)	0	0.06	2.02 (16.4)	0.10	0.08
	PAD	ns	−60 **	ns	102	5.9 (5.8)	0	0.09	5.44 (6)	0.09	0
Negative ankle moment (Nm∙kg^−1^)	Healthy	ns	154.5 **	−1593 **	98.2	5.8 (11.5)	0.12	0.04	8.5 (21)	0	0.06
	PAD	ns	ns	−2084 *	100	12.4 (18.8)	0.08	0.05	4.6 (14.3)	0	0.09
Positive ankle power (W·kg^−1^)	Healthy	ns	78.5 **	ns	99	2.4 (4.5)	0.07	0	7.5 (9.1)	0.07	0.09
	PAD	56.3 *	66.7 **	ns	98	0.8 (5.1)	0	0.06	5.8 (5.7)	0.08	0.05
Negative ankle power (W·kg^−1^)	Healthy	89 **	ns	−1437 **	97	7.7 (6.5)	0	0.10	6.2 (9.7)	0.12	0
	PAD	ns	ns	ns	102	0.4 (5)	0.06	0	7 (6.6)	0.09	0
Mean soleus activity (%)	Healthy	ns	ns	1143 **	100	1.1 (7.2)	0	0.06	10.3 (13)	0.10	0.08
	PAD	70 *	ns	ns	97	3.6 (5.3)	0	0.06	8.4 (9)	0.09	0
Mean gastrocnemius medialis activity (%)	Healthy	73 *	ns	ns	96	11.8 (13)	0	0.06	8.05 (14.4)	0.12	0
	PAD	ns	−145*	ns	107	7.15 (5.8)	0	0.09	14 (29)	0.05	0.05
Positive knee moment (Nm∙kg^−1^)	Healthy	−107 *	ns	2507 **	107	14.1 (17.6)	0.12	0	20 (8.7)	0.1	0.08
	PAD	ns	ns	ns	101	8.5 (19.5)	0.09	0	9.8 (4.7)	0	0.09
Negative knee moment (Nm∙kg^−1^)	Healthy	ns	ns	ns	97	7.7 (9.2)	0.07	0	4.2 (17.1)	0.12	0
	PAD	ns	ns	ns	99	7.7 (7.0)	0	0.09	8.8 (17.8)	0.09	0
Positive knee power (W·kg^−1^)	Healthy	ns	ns	ns	99	14.8 (40)	0	0.10	8.5 (29.4)	0	0.06
	PAD	−134 *	ns	4056 **	91	24.3 (9.3)	0.09	0	4.2 (19.5)	0.08	0.05
Negative knee power (W·kg^−1^)	Healthy	−51 *	ns	1244.5 **	101	6.0 (5.9)	0.12	0	7.6 (8.2)	0.1	0.08
	PAD	ns	ns	ns	103	0.5 (4.0)	0.0950	0	6.3 (6.6)	0.05	0.05
Mean vastus medialis activity (%)	Healthy	ns	165 *	−1822 *	106	0	0	0	22.8 (32.6)	0.07	0.09
	PAD	ns	222 **	ns	91	16.2 (5.2)	0.09	0	10.3 (16)	0	0.09
Mean tibialis anterior activity (%)	Healthy	ns	ns	ns	102	4.3 (19)	0	0.06	7.78 (9.5)	0	0.1
	PAD	ns	ns	ns	99	6.15 (5)	0	0.06	9.5 (18)	0.08	0.05
